# Influence of Differing Analgesic Formulations of Aspirin on Pharmacokinetic Parameters

**DOI:** 10.3390/pharmaceutics7030188

**Published:** 2015-08-03

**Authors:** Kunal Kanani, Sergio C. Gatoulis, Michael Voelker

**Affiliations:** 1 School of Pharmacy, Rutgers, The State University of New Jersey, 160 Frelinghuysen Road, Piscataway, NJ 08854, USA; E-Mail: kananikunal@gmail.com; 2 Bayer HealthCare, 100 Bayer Blvd, Whippany, NJ 07981, USA; 3 Bayer HealthCare, Building K56, 51368 Leverkusen, Germany; E-Mail: michael.voelker@bayer.com

**Keywords:** clinical pharmacokinetics, pharmacokinetics, absorption, formulation, prodrugs, aspirin

## Abstract

Aspirin has been used therapeutically for over 100 years. As the originator and an important marketer of aspirin-containing products, Bayer’s clinical trial database contains numerous reports of the pharmacokinetics of various aspirin formulations. These include evaluations of plain tablets, effervescent tablets, granules, chewable tablets, and fast-release tablets. This publication seeks to expand upon the available pharmacokinetic information concerning aspirin formulations. In the pre-systemic circulation, acetylsalicylic acid (ASA) is rapidly converted into its main active metabolite, salicylic acid (SA). Therefore, both substances are measured in plasma and reported in the results. The 500 mg strength of each formulation was chosen for analysis as this is the most commonly used for analgesia. A total of 22 studies were included in the analysis. All formulations of 500 mg aspirin result in comparable plasma exposure to ASA and SA as evidenced by AUC. Tablets and dry granules provide a consistently lower *C*_max_ compared to effervescent, granules in suspension and fast release tablets. Effervescent tablets, fast release tablets, and granules in suspension provide a consistently lower median *T*_max_ compared to dry granules and tablets for both ASA and SA. This report reinforces the importance of formulation differences and their impact on pharmacokinetic parameters.

## 1. Introduction

Aspirin (acetylsalicylic acid, ASA) has been used therapeutically for over 100 years. As a salicylate derivative, it possesses the three properties of non-steroidal anti-inflammatory drugs (NSAIDs): analgesic, anti-pyretic, and anti-inflammatory actions. The principal therapeutic effect of NSAIDs, such as aspirin, is their ability to inhibit prostaglandin production [1]. The first enzyme in the prostaglandin synthetic pathway is prostaglandin endoperoxide synthase (PGHS), or cyclooxygenase (COX). COX catalyzes the conversion of arachidonic acid to prostaglandin H2 (PGH2), which is a precursor to other prostaglandins. COX exists in two isoforms: COX-1 and COX-2. In general COX-1 is the constitutive form, whereas COX-2 is an inducible isoform. Aspirin covalently modifies both COX-1 and COX-2, thus resulting in an irreversible inhibition of cyclooxygenase activity, unlike other NSAIDs that bind to COX reversibly [2]. Inhibition of COX-1 leads to inhibition of platelet aggregation, but also causes irritation of gastric lining and kidney function. Inhibition of COX-2 leads to anti-inflammatory, antipyretic and analgesic actions.

The absorption of aspirin when administered orally is rapid and complete. The absorption is directly proportional to the dose and, thus, follows first order kinetics. Absorption takes place, to a minor extent, in the stomach and, to a larger extent, in the upper small intestine [3]. The bioavailability of most drug substances in the gastrointestinal lumen is greater when the drug is in the non-ionized state. Only the non-dissociated molecule is sufficiently lipophilic to be able to pass through the gastric or intestinal wall by passive diffusion. Aspirin, being a weak acid (pKa 3.5), is dissociated to a greater extent in the intestine owing to the higher pH in that part of the gastrointestinal (GI) tract, thus improving its solubility. This phenomenon, coupled with a larger surface area, results in higher total absorption from this part of the GI tract, despite the higher degree of dissociation [3].

As a result of the various therapeutic uses of aspirin and salicylic acid (SA), which have included the treatment of mild to moderate pain and fever, reduction in risk of chronic diseases such as thrombotic cardiovascular events and, as suggested by emerging evidence, the reduction in risk of colorectal cancer, aspirin has taken many shapes and sizes [4,5,6,7,8,9]. Although aspirin was first synthesized in 1897 and sold as a tablet for many decades, over the years dosage forms have included plain tablets, chewable tablets, effervescent tablets, extended-release tablets, granules and suspensions, fast release/disintegrating tablets, powders, creams, lotions, and intravenous solutions.

These dosage forms are meant to complement and enhance the pharmacokinetic (PK) and pharmacodynamic actions of aspirin for the given indication, and therefore may have an impact on efficacy and safety. For the treatment of pain and fever, these enhancements typically include bioavailability (total exposure of drug measured as area under the plasma-concentration time curve [AUC]), reduction in the time to reach maximum plasma concentration (*T*_max_), increase in maximum plasma concentration (*C*_max_), and extension of the release profile over time. For instance, modifications to a plain tablet by increasing the rate of tablet dissolution can reduce the time of absorption and therefore shorten the time to onset of a clinical effect.

Published product-specific pharmacokinetic information on aspirin is limited. As the originator and an important marketer of aspirin-containing products, Bayer’s clinical trial database contains numerous reports of the pharmacokinetics of various aspirin formulations. These include evaluations of plain tablets, effervescent tablets, granules, chewable tablets, and fast-release tablets. While some of these data are available in the literature, the authors are aware of no comprehensive overview of the pharmacokinetics of these aspirin formulations [10,11,12,13]. Knowledge of the importance of formulation differences on clinical effect led to the development of a fast-release tablet, designed to improve the analgesic speed of onset. The data characterizing the performance of this formulation have been published previously [10].

The fast-release tablet combines two formulation enhancements to achieve this: a reduction in particle size, thereby increasing the available surface area for absorption, and the addition of sodium carbonate, which acts as both a disintegrant and local buffer. The granules are solid dosage forms that are composed of agglomerations of smaller particles. These multi-component compositions are prepared for oral administration and are used to facilitate flexible dosing regimens as granules or as suspensions, address stability challenges, allow taste masking, or facilitate flexibility in administration. Effervescent tablets are prepared by compaction and contain, in addition to the active pharmaceutical ingredient(s), mixtures of acids (e.g., citric acid or tartaric acid) and carbonates and/or hydrogen carbonates. Upon contact with water, these formulations release carbon dioxide, producing the characteristic effervescent action. Chewable tablets are compressed dosage forms that are chewed in the mouth prior to swallowing. This formulation typically contains sweeteners, flavors, and taste masking agents to make the dosage more palatable.

This publication seeks to expand upon the available pharmacokinetic information concerning immediate release aspirin formulations, including plain tablets, effervescent tablets, granules, chewable tablets, and fast-release tablets. Extended release formulations are not the subject of this review.

## 2. Experimental Section

The Bayer internal database contains over 60 multi-arm, multi-period aspirin pharmacokinetic studies. These studies were conducted in healthy volunteers and were randomized, open-label, single dose studies ranging from 40 to 1000 mg at various centers throughout the world. The studies were conducted in accordance with the Declaration of Helsinki and ICH GCP standards. The studies recruited healthy patients, male or female (18–65 years of age) with a PK evaluable population of 20–30 patients per study. Prior to enrolment, the patients gave their informed consent in writing. Included studies were conducted between year 1991 and 2011 in the United States, Germany, or Japan. High performance liquid chromatography (HPLC) methods used for the determination of ASA and SA plasma concentrations were developed and validated by the bioanalytical laboratories.

The current analysis comprises 22 studies performed under fasting conditions, although studies may have contributed data on more than one aspirin formulation. The doses were determined by the indication for the product, e.g., pain/fever or platelet inhibition. For the purposes of this review, we have included studies of aspirin at 500 mg because this is the most common strength for symptomatic treatment of pain, fever, and common cold. Individual studies present the full range of pharmacokinetic parameters, however for the purpose of this analysis we concentrate on the most relevant parameters *C*_max_, AUC_0-inf_ and *T*_max_.

These pharmacokinetic parameters discussed are defined as follows:

- *C*_max_ = Maximum drug concentration in plasma after single dose administration;
- AUC_0-inf_ = Area under the plasma concentration *vs.* time curve from zero to infinity after single dose;
- *T*_max_ = Time to reach maximum drug concentration in plasma after single dose.

All data were extracted from individual clinical study reports for each study. Given that data were collected over a decade of clinical studies spanning multiple countries, only these pharmacokinetic parameters have been discussed to uniformly analyze all the formulations. Each study result was grouped by formulation and data were then analyzed using descriptive and inferential statistics including mean, median, and their 95% confidence intervals. Data in the tables are reported as geometric mean ± standard deviation for *C*_max_, and AUC. Confidence Intervals (95% CI) for the mean are reported for *C*_max_ and AUC and 95% CI for the median is reported for *T*_max_ for all formulations (except chewable tablets).

In the pre-systemic circulation, acetylsalicylic acid is rapidly converted into its main active metabolite, salicylic acid. Consequently, in aspirin pharmacokinetic studies both substances (ASA and SA) are measured in plasma and reported in results and consequently discussed in this report.

## 3. Results and Discussion

The following tables (Table 1 and Table 2) provide a summary of pharmacokinetic data of acetylsalicylic acid and salicylic acid for all the aspirin formulations mentioned above.

A total of 22 studies were included in the analysis. The remaining studies from the database have not been included because the formulation is not relevant to this report (e.g., IV formulation, 81 mg formulation). Aspirin tablets have the most number of studies included in the analysis because it is the most commonly used formulation and other dosage forms were assessed for bioavailability in comparison to the plain tablet.

**Table 1:** Summary pharmacokinetic data of acetylsalicylic acid for aspirin formulations.

Formulation	No. of studies	*C*_max_ (mg/L)	AUC [(mg × h)/L]	*T*_max_ (h)
Aspirin tablet	10	Mean: 5.43 ± 1.38 95% CI: 4.66–6.21 Median: 5.69 ± 1.38	Mean: 6.21 ± 1.24 95% CI: 5.51–6.91 Median: 6.22 ± 1.24	Median: 0.5 ± 0.16 95% CI: 0.40–0.60
Aspirin effervescent tablet	3	Mean: 10.45 ± 1.18 95% CI: 9.12–11.78 Median: 11.08 ± 1.18	Mean: 5.27 ± 0.51 95% CI: 4.69–5.85 Median: 5.31 ± 0.51	Median: 0.33 ± 0.02 95% CI: 0.30–0.36
Aspirin granules	6	Mean: 5.42 ± 1.03 95% CI: 4.59–6.25 Median: 5.48 ± 1.03	Mean: 6.18 ± 1.36 95% CI: 5.09–7.27 Median: 5.97 ± 1.36	Median: 0.46 ± 0.13 95% CI: 0.36–0.56
Aspirin granules in suspension	6	Mean: 12.77 ± 1.94 95% CI: 11.43–14.11 Median: 12.69 ± 1.94	Mean: 6.77 ± 1.63 95% CI: 5.64–7.90 Median: 6.02 ± 1.63	Median: 0.25 ± 0.04 95% CI: 0.22–0.28
Fast release aspirin tablet	6	Mean: 13.89 ± 1.08 95% CI: 13.03–14.77 Median: 13.75 ± 1.08	Mean: 6.95 ± 0.67 95% CI: 6.42–7.48 Median: 6.84 ± 0.66	Median: 0.30 ± 0.02 95% CI: 0.28–0.32
Aspirin chewable tablet	2	Mean: 6.25 ± 0.24 95% CI: 5.92–6.58 Median: 6.25 ± 0.24	Mean: 4.67 ± 0.03 95% CI: 4.63–4.71 Median: 4.67 ± 0.03	Median: 0.33 ± 0 95% CI: n/a

**Table 2:** Summary pharmacokinetic data of salicylic acid for aspirin formulations.

Formulation	No. of studies	*C*_max_ (mg/L)	AUC [(mg × h)/L]	*T*_max_ (h)
Aspirin tablet	10	Mean: 25.45 ± 3.64 95% CI: 23.19–27.71 Median: 25.56 ± 3.64	Mean: 145.67 ± 35.24 95% CI:123.82–167.50 Median: 125.76 ± 35.24	Median: 2.00 ± 0.54 95% CI: 1.66–2.34
Aspirin effervescent tablet	3	Mean: 27.54 ± 1.18 95% CI: 26.20–28.88 Median: 27.31 ± 1.18	Mean: 138.07 ± 8.89 95% CI: 128.00–148.13 Median: 141.90 ± 8.89	Median: 0.75 ± 0.05 95% CI: 0.70–0.80
Aspirin granules	6	Mean: 25.51 ± 4.59 95% CI: 21.84–29.18 Median: 25.50 ± 4.59	Mean: 158.4 ± 50.50 95% CI: 118.00–198.80 Median: 156.00 ± 50.50	Median: 2.00 ± 0.54 95% CI: 1.56–2.44
Aspirin granules in suspension	6	Mean: 29.08 ± 2.66 95% CI: 27.31–31.95 Median: 29.64 ± 2.66	Mean: 132.54 ± 16.22 95% CI: 121.30–143.78 Median: 133.51 ± 16.22	Median: 0.83 ± 0.15 95% CI: 0.72–0.94
Fast release aspirin tablet	6	Mean: 31.80 ± 1.81 95% CI: 30.35–33.25 Median: 31.00 ± 1.81	Mean: 179.07 ± 15.27 95% CI: 166.85–191.29 Median: 182.15 ± 15.27	Median: 0.75 ± 0.05 95% CI: 0.71–0.79
Aspirin chewable tablet	2	Mean: 23.24 ± 1.17 95% CI: 21.62–24.86 Median: 23.24 ± 1.17	Mean: 123.18 ± 0.24 95% CI: 122.85–123.51 Median: 123.18 ± 0.24	Median: 1.25 ± 0 95% CI: n/a

**Figure 1:**
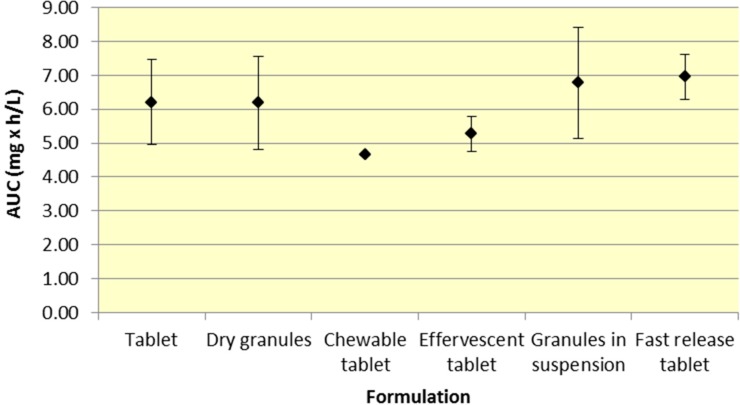
Summary ASA AUC for various 500 mg formulations (Mean ± SD).

**Figure 2:**
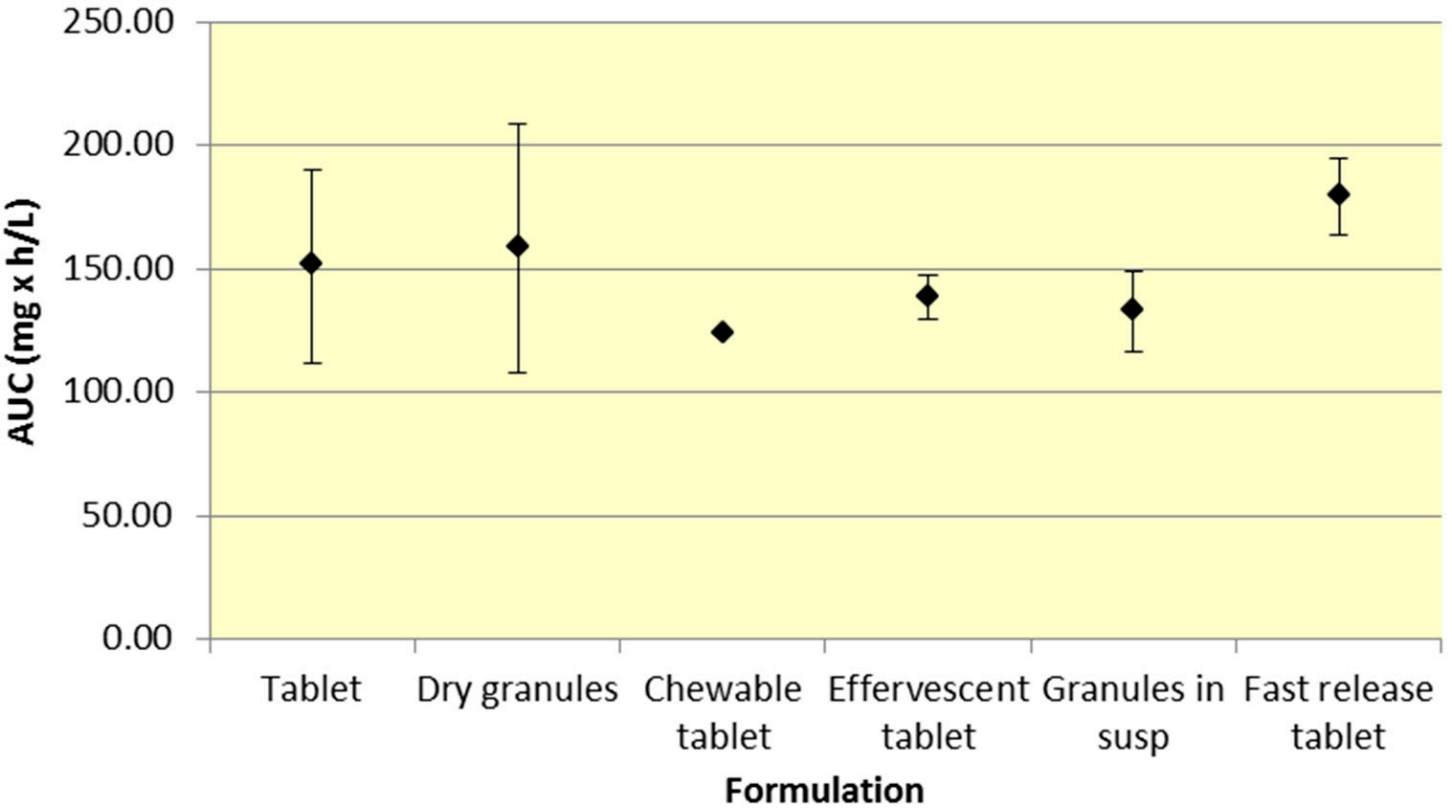
Summary SA AUC for various 500 mg formulations (Mean ± SD).

All formulations of 500 mg aspirin result in comparable plasma exposure to ASA and SA as evidenced by AUC. The 95% CIs for both ASA and SA overlap to a significant extent, indicating comparable overall exposure regardless of formulation (Figure 1 and Figure 2, Table 1 and Table 2).

**Figure 3:**
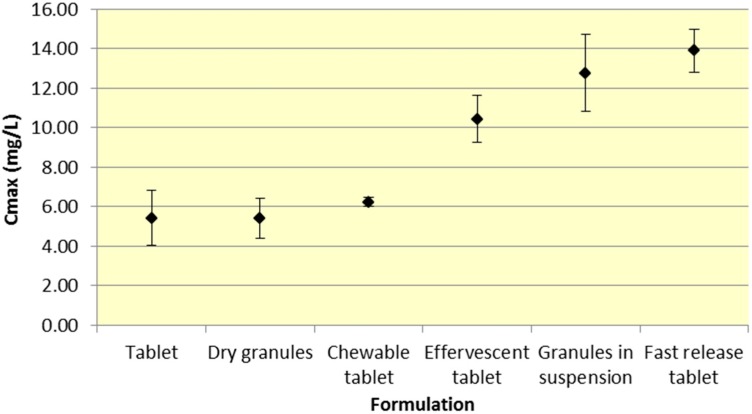
Summary ASA *C*_max_ for various 500 mg formulations (Mean ± SD).

**Figure 4:**
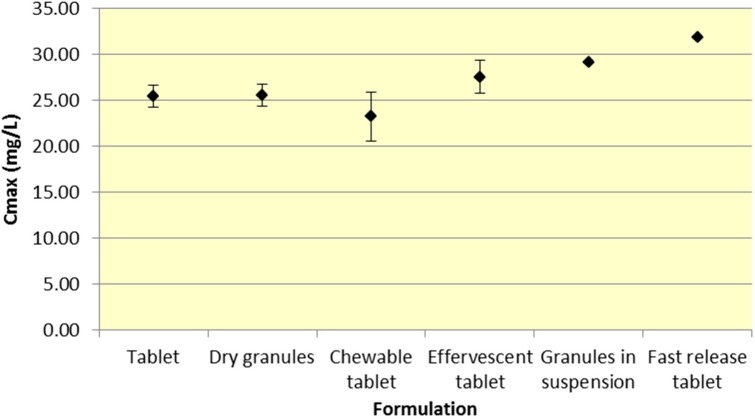
Summary SA *C*_max_ for various 500 mg formulations (Mean ± SD).

Tablets and dry granules provide a consistently lower *C*_max_ compared to effervescent tablets, granules in suspension, and fast release tablets. However, the differences in *C*_max_ are more pronounced for ASA versus SA. Furthermore, chewable tablets produce a mean *C*_max_ that is higher than tablet or dry granules for ASA but produce the lowest *C*_max_ for ASA (Figure 3 and Figure 4, Table 1 and Table 2).

**Figure 5:**
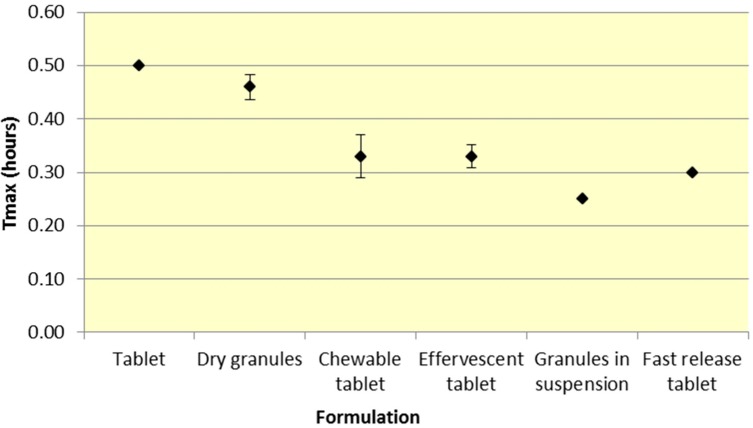
Summary ASA *T*_max_ for various 500 mg formulations (Median ± SD).

**Figure 6:**
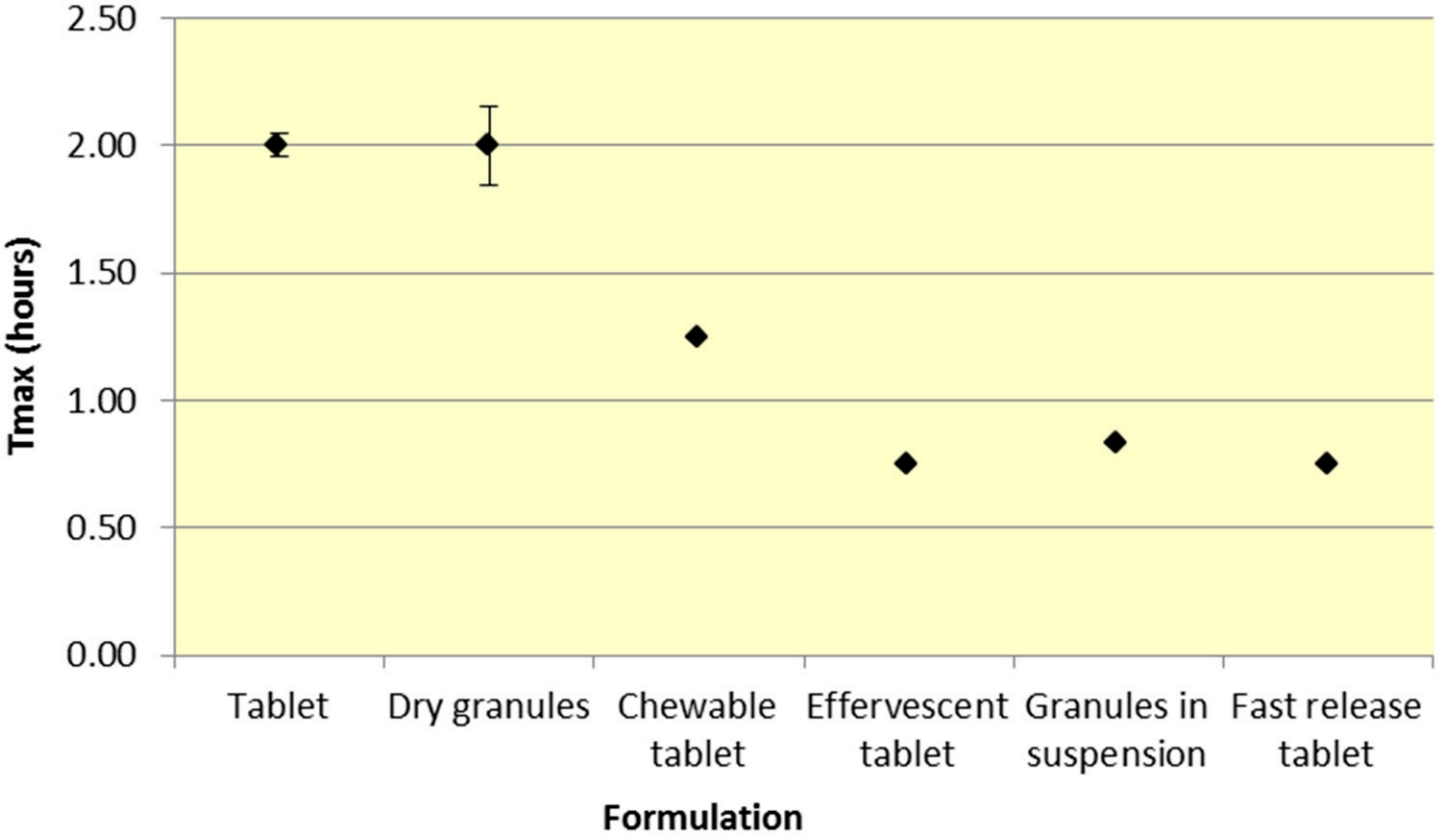
Summary SA *T*_max_ for various 500 mg formulations (Median ± SD).

Similar to *C*_max_, effervescent tablets, fast release tablets and granules in suspension provide a consistently lower median *T*_max_ compared to dry granules and tablets for both ASA and SA (Figure 5 and Figure 6, Table 1 and Table 2).

The results presented above provide insight into the pharmacokinetic profiles of many common aspirin formulations. AUC reflects the extent of drug absorption and *C*_max_ and *T*_max_ are important features of the plasma profile, providing an insight into rate of absorption, peak effect and onset of action.

Although a majority of the studies have incorporated aspirin plain tablets (the most common formulation for aspirin), it is still possible to analyze differences among the various aspirin formulations. As seen in Figure 1 and Figure 2, there was comparable exposure of both ASA and SA for all 500 mg formulations. Although various formulations provide different *C*_max_ and *T*_max_ profiles, the total exposure remained reasonably constant, as indicated by the overlapping 95% CIs for AUC. The comparable level of systemic exposure suggests that the safety and efficacy profiles of the products may be similar. Given that fast release tablets are weakly ionized, based on the data, the bioavailability would be expected to be greatest for the fast release tablet formulation compared to granules (in suspension) or effervescent tablets, all of which exhibit greater buffering capacity. The fast release tablets rapidly dissolve and do not raise the pH of the stomach significantly, compared to other formulations leading to slightly higher bioavailability.

As seen in Figure 3, *C*_max_ levels for tablet and dry granule formulations are considerably lower than effervescent, granules in suspension, and fast release tablets for ASA. The latter formulations with the exception of fast release tablet (discussed above) are in liquid form and therefore highly bioavailable from solution and therefore have a faster absorption time. Additionally, effervescent aspirin tablets promote gastric emptying by promoting stomach motility, which may help enhance the onset of action [14]. On the other hand, aspirin dry granules, chewable tablets, and plain tablets have to first be dissolved in solution, after which they can be absorbed systemically, thereby increasing *T*_max_ and lowering *C*_max_.

Although this review seeks to provide a summary of the pharmacokinetic parameters of various aspirin formulations, it is important to note that this data shows marked variability. Reasons for variability among studies include inter-patient variability, inter-study variability, formulation, and analytical variability. The studies presented in this review have been conducted in many centers throughout the world and therefore “center effect” has to be considered, given different operating procedures and practices among various centers. Demographic differences may also partly explain differences in results as absorption, metabolism, and excretion of ASA and SA can vary among subjects. Furthermore, the samples have been analyzed over the years at various analytical centers using different technology and methods which may have led to variability among summary results as seen from the standard deviations for each formulation.

Notably, all studies were performed in a fasting state in healthy volunteers. A recently published review compares the difference in pharmacokinetic parameters in fed versus fasted state for common analgesics [15]. The evaluation of the effect of food on pharmacokinetic parameters was not the subject of this review, although it remains an important issue, particularly for OTC drugs.

## 4. Conclusions

The present report expands upon the available pharmacokinetic information concerning aspirin formulations for pain and fever, including plain tablets, effervescent tablets, granules, chewable tablets, and fast-release tablets. It reinforces the importance of formulation differences and their impact on pharmacokinetic parameters. Techniques such as increasing solubility, degree of ionization, and surface area can have a significant impact on aspirin pharmacokinetics, which has been demonstrated to reduce the time to clinical onset of analgesia. The availability of various analgesic formulations of aspirin provides clinicians and consumers with different options depending on consumer preference and the characteristics of pain. Acute painful conditions may be effectively managed by rapidly-acting formulations such as fast release tablets and effervescent tablets, where the onset of analgesia is an important consideration.
